# CB_2_ Cannabinoid Receptor As Potential Target against Alzheimer's Disease

**DOI:** 10.3389/fnins.2016.00243

**Published:** 2016-05-31

**Authors:** Ester Aso, Isidro Ferrer

**Affiliations:** ^1^Institut de Neuropatologia, Servei d'Anatomia Patològica, Bellvitge Biomedical Research Institute (IDIBELL)-Hospital Universitari de Bellvitge, Universitat de BarcelonaL'Hospitalet de Llobregat, Spain; ^2^CIBERNED - Centro de Investigación Biomédica en Red de Enfermedades Neurodegenerativas, Instituto Carlos IIIMadrid, Spain

**Keywords:** CB_2_ receptor, cannabinoids, Alzheimer, neuroinflammation, β-amyloid, tau, oxidative stress

## Abstract

The CB_2_ receptor is one of the components of the endogenous cannabinoid system, a complex network of signaling molecules and receptors involved in the homeostatic control of several physiological functions. Accumulated evidence suggests a role for CB_2_ receptors in Alzheimer's disease (AD) and indicates their potential as a therapeutic target against this neurodegenerative disease. Levels of CB_2_ receptors are significantly increased in *post-mortem* AD brains, mainly in microglia surrounding senile plaques, and their expression levels correlate with the amounts of Aβ_42_ and β-amyloid plaque deposition. Moreover, several studies on animal models of AD have demonstrated that specific CB_2_ receptor agonists, which are devoid of psychoactive effects, reduce AD-like pathology, resulting in attenuation of the inflammation associated with the disease but also modulating Aβ and tau aberrant processing, among other effects. CB_2_ receptor activation also improves cognitive impairment in animal models of AD. This review discusses available data regarding the role of CB_2_ receptors in AD and the potential usefulness of specific agonists of these receptors against AD.

## Overview of Alzheimer's disease

Alzheimer's disease (AD) is an age-dependent neurodegenerative disorder characterized by slowly progressive cognitive decline with fatal outcome. To date, no effective treatment is available. Dementia due to AD occurs in one in nine people aged 65 and in about one in four at the age of 85 (Hebert et al., 2013). Prevalence is expected to grow in coming decades as the size and proportion of the older population continue to increase due to the rise in life expectancy in developed countries (Hebert et al., 2013).

AD is morphologically distinguished by the presence in the brain of senile plaques, mainly composed of different species of fibrillar β-amyloid (Aβ) produced by the cleavage of the β-amyloid precursor protein (APP), and neurofibrillary tangles composed of various isoforms of hyper-phosphorylated and truncated tau protein. Senile plaques are surrounded by dystrophic neurites, reactive astrocytes, and microglia. Neurofibrillary tangles first appear in selected nuclei of the brain stem, and entorhinal and transentorhinal cortex, and then progress to the hippocampus and limbic system, and finally to most of the telencephalon (Braak and Braak, 1991). The development and progression of senile plaques does not parallel the evolution of tau pathology in sporadic AD (Thal et al., 2002). Aβ and tau misfolded proteins compromise neural activity due to an increase in toxic function and/or loss of their normal function, thus contributing to the decline of neuronal organization manifested as synaptic dysfunction and neuronal death (Duyckaerts and Dickson, 2011; Ferrer, 2012). A self-propagating process of misfolded proteins has been suggested to explain disease progression (Jucker and Walker, 2013). Aβ and misfolded tau aggregate into seeds that are able to modify native proteins causing them to aggregate and to form pathogenic assemblies in a prion-like way (Meyer-Luehmann et al., 2006; Clavaguera et al., 2009; Stöhr et al., 2012).

It is important to note that AD-related pathology begins more than 20 years before the onset of dementia. First stages of AD in which lesions are restricted to the brain stem and inner parts of the temporal lobe are usually asymptomatic. About 80% of individuals aged 65 present senile plaques and/or neurofibrillary tangles in specific brain areas but only about 5% of them suffer from dementia. This is an important point as AD-related pathology is common in the elderly but this does not inevitably lead to dementia. AD changes restricted to the inner temporal lobe can progress slowly and be well tolerated in some individuals. Only the accumulation of lesions in certain individuals determines a progression of the neurodegenerative disease, which leads to dementia once reached determinate threshold (Ferrer, 2012). The slow progression of the neurodegenerative process visualizes a putative temporal window for therapeutic intervention. However, to date most therapeutic interventions aimed at modifying a single pathological factor (e.g., cholinergic dysfunction, inflammation, Aβ and/or tau aberrant processing) have failed because of their limited benefit or for safety reasons (Scheltens et al., 2016). Considering that multiple alterations are concomitant to Aβ and tau aberrant processing in AD (Ferrer, 2012), compounds with pleiotropic activity which will target in parallel several processes that play key roles in AD are expected to yield greater benefits than those obtained by current therapies (Bolognesi et al., 2009; Frautschy and Cole, 2010). Inflammation, mitochondrial dysfunction, oxidative stress, and impaired function of degradation pathways are the most prominent concomitant pathological events (Keller et al., 2000; Ferrer, 2009; Sultana and Butterfield, 2010; López-González et al., 2015), as briefly described in the following paragraphs. These alterations are potential targets of therapeutic intervention.

Inflammation has been proposed as a key factor in the pathogenesis of AD. This is characterized by microglial activation, reactive astrocytes and elevated expression of cytokines and mediators of the inflammatory response. It has been proposed that microglial activation in AD can have beneficial and detrimental effects depending on the stage of the disease. Thus, the acute microglial reaction aims at removing the abnormal protein aggregates appearing at the early stages of the disease. However, cumulative formation of aberrant protein aggregates drives to chronic inflammation which has detrimental consequences due to the sustained exposure to chemokines, cytokines and other inflammatory mediators (Heneka et al., 2015). Conversion of microglia from detrimental (M1) to beneficial (M2) phenotype may be achieved by modulation of pro-inflammatory signaling pathways such as the NLRP3 inflammasome (Heneka et al., 2013). Similar to microglial cells, astrocytes contribute to inflammation in AD by releasing cytokines, interleukins, nitric oxide (NO), and other toxic molecules in response to Aβ exposure at the time they also participate in the internalization and degradation of Aβ (Heneka et al., 2015). However, anti-inflammatory treatments failed to produce beneficial effects in patients with severe cognitive impairment and dementia. This fact is probably due to the fact that inflammatory responses in AD differ not only depending on the stage of the disease but also on the region involved (López-González et al., 2015). That means that inflammatory responses in some regions have a beneficial phenotype whereas they have a deleterious phenotype in other regions in the same individual, thus stressing the need to identify new regulators or modulators of the inflammatory response that can be adapted to specific molecular targets (López-González et al., 2015).

Altered mitochondria are also key factors in the pathogenesis of AD. This includes impaired energy metabolism and increased production of free radicals with subsequent oxidative and nitrosative damage affecting lipids, proteins and nucleic acids (Sultana and Butterfield, 2010). These alterations are already observed in the entorhinal cortex at early stages of AD ultimately leading to neuron exhaustion (Ferrer, 2009). Several studies in AD transgenic mouse models support the potential beneficial effect of compounds targeting mitochondrial dysfunction although the clinical benefit of such drugs in humans is still not known (Onyango et al., 2016).

Finally, another prominent concomitant pathological event in AD is impaired function of degradation pathways (Keller et al., 2000). Oxidative damage and some other pathological events may alter protein structure and function in AD. These modified proteins have to be removed to prevent their toxic accumulation. However, the ubiquitin-proteasome system and autophagy mechanisms are impaired due to the toxic effects of Aβ and oxidative stress damage thus leading to the accumulation of oxidized/unfolded proteins that may contribute to neuronal loss (Tramutola et al., 2016).

## Endogenous cannabinoid system: a role in neurodegenerative diseases

Among the candidates to fulfill the requirements for novel effective multi-target therapies against neurodegenerative diseases are newly emerging compounds that target the endogenous cannabinoid system (ECS; Aso and Ferrer, 2014; Fagan and Campbell, 2014; Fernández-Ruiz et al., 2015). Interest in the ECS derives from the pleiotropic activity of this complex network of lipid molecules and receptors, which is involved in homeostatic control of several physiological functions in brain and other organs (Iannotti et al., 2016). The ECS is composed of (i) at least two subtypes of cannabinoid G_i∕o_-coupled receptors, CB_1_ and CB_2_ (Pertwee et al., 2010), (ii) certain endogenous ligands, mainly arachidonoylethanolamine or anandamide (AEA) and 2-arachidonoylglycerol (2-AG) derived from the membrane phospholipids (Pertwee, 2015), (iii) several enzymes responsible for endocannabinoid biosynthesis and metabolism (Ligresti et al., 2005), and (iv) molecules linked to the cellular uptake and transport of certain endocannabinoids (Fowler, 2013). CB_1_ receptors are the most abundant cannabinoid receptors and are located in brain, mainly in neurons but also in glial cells, and in peripheral tissues (Hu and Mackie, 2015). CB_1_ activity regulates important brain functions including cognition and memory, emotion, motor control, feeding, and pain perception, by modulating excitatory and inhibitory neurotransmission (Wilson and Nicoll, 2002; Howlett, 2005). Moreover, CB_1_ receptors mediate psychoactive effects of cannabis derivatives (Maldonado et al., 2011). In contrast, activation of CB_2_ receptors is not accompanied by psychoactive effects (Buckley et al., 2000). CB_2_ was initially considered a peripheral cannabinoid receptor because *in situ* hybridization analysis revealed high levels of CB_2_ mRNA in spleen but levels below the detection thresholds in brain. CB_2_ receptors were demonstrated to modulate immune cell migration and the release of cytokines in cells of the immune system (Cabral and Griffin-Thomas, 2009). However, more recent findings have shown that CB_2_ receptors are also present in other tissues including the central nervous system (Atwood and Mackie, 2010). CB_2_ receptors are highly inducible and under certain conditions are expressed in brain, mainly by microglia, with levels increasing as these immune cells are activated. CB_2_ modulates microglial migration and infiltration into brain areas with active neuroinflammation and degeneration (Walter et al., 2003; Fernández-Ruiz et al., 2008). Moreover, CB_2_ receptors are also present at detectable and functional levels in a subset of neurons with increasing expression levels following injury (Atwood and Mackie, 2010). Apart from the regulation of inflammatory processes, some experimental designs also suggest that CB_2_ receptors may play a role in nociception (Jhaveri et al., 2007; Whiteside et al., 2007), gastrointestinal function (Wright et al., 2008), neural progenitor cell proliferation and axon guidance (Palazuelos et al., 2012; Duff et al., 2013), and synaptic transmission (Kim and Li, 2015; Li and Kim, 2016), among other functions. Most of the evidence comes from pharmacological studies using specific CB_2_ agonists and antagonists, and from genetically manipulated mice. However, the location of CB_2_ receptors mediating such effects is not conclusively documented.

As mentioned before, the ECS has a pleiotropic activity and is able to modulate several alterations occurring during normal and pathological aging, including protein misfolding, inflammation, excitotoxicity, mitochondrial dysfunction, and oxidative stress (Bilkei-Gorzo, 2012; Aso and Ferrer, 2014; Fagan and Campbell, 2014; Fernández-Ruiz et al., 2015). Evidence about the role of ECS on aging derives from observations in genetic models. Thus, deficiency in CB_1_ receptors contributes to acceleration of aging (Bilkei-Gorzo et al., 2005, 2012) whereas deletion of the endocannabinoid degrading enzyme FAAH enhances age-related microglial activity and concomitant inflammatory responses in brain (Ativie et al., 2015). In contrast, stimulation of certain ECS components produces beneficial effects in experimental models of neurodegenerative diseases (Aso and Ferrer, 2014; Fagan and Campbell, 2014; Fernández-Ruiz et al., 2015). These findings demonstrate a role for ECS in normal and pathological aging that has sustained interest in developing therapies against neurodegenerative diseases based on ECS modulation. Major attention has been focused on the use of cannabinoid agonists, but the psychoactive effects elicited by compounds targeting CB_1_ receptors have served to limit their potential development in clinical practice. For this reason, the study of specific CB_2_ agonists which are devoid of psychoactive effects is promising, although detailed clinical evaluation is still needed (Atwood et al., 2012).

## CB_2_ receptors in AD brains

A few studies have addressed the analysis of CB_2_ contents in AD brain but all of them have resulted in similar findings. A significant increase in CB_2_ receptor levels has been found in *post-mortem* AD brains mainly expressed in microglia surrounding senile plaques (Benito et al., 2003; Ramírez et al., 2005; Solas et al., 2013). Similarly, enhanced CB_2_ PET binding has been reported in the brain in an animal model of AD (Savonenko et al., 2015). In addition, CB_2_-specific staining is also observed in tangle-like bearing neurons and in dystrophic neurites from frontal cortex in AD (Ramírez et al., 2005). Interestingly, expression levels of CB_2_ receptors correlate with Aβ_42_ levels and plaque deposition although not with cognitive status (Solas et al., 2013), thus suggesting that these pathogenic events induce CB_2_ receptor expression. The strong induction of CB_2_ receptors in affected microglia is therapeutically advantageous since it would permit their selective activation in damaged tissues, thereby minimizing the possibility of deleterious side effects. However, CB_2_ receptors in AD brain are nitrosylated, probably as a consequence of microglial activation and peroxynitrite radical formation, and this may contribute to the impaired coupling of these receptors to downstream effector signaling molecules (Ramírez et al., 2005). Nevertheless, the functionality of CB_2_ receptors seems to be at least partially preserved in AD according to the results of pharmacological experiments carried out in AD models, as described in the following sections.

## CB_2_ receptor as a therapeutic target in AD: evidence from experimental models

During the last decade, a number of studies have provided experimental evidence about the potential therapeutic properties of compounds targeting CB_2_ receptors in cellular and animal models that mimic a variety of AD-related changes. A summary of pharmacological findings supporting this hypothesis is shown in Table 1. Moreover, at least three different genetically manipulated murine models have recently been created to further demonstrate a role for CB_2_ receptors in this neurodegenerative disease (Table 2). Most of these assays are focused on the potential benefit derived from the well-known anti-inflammatory properties of CB_2_ agonists, but some of them also reveal the capacity of CB_2_ receptors to modulate Aβ and hyper-phosphorylated tau levels, among other molecular alterations.

**Table 1:** **Pharmacological evidence of CB_**2**_ receptor as a therapeutic target in AD**.

**References**	**AD model**	**Compound acting on CB_2_ receptors**	**CB_2_-mediated effect**
Ehrhart et al., 2005	Aβ_1−42_	JWH-015	↓ IFN-γ-mediated CD40 expression
	Microglial cells culture		↓ TNF-α production
			↑ Phagocytosis of Aβ
			↓ NO
Ramírez et al., 2005	Aβ_25−35_ and Aβ_1−40_	HU-210	↑ Neuronal survival
	Microglial rat primary culture	WIN55,212-2	↓ Microglial reactivity to Aβ
	Neuronal rat primary culture	JWH-133	↓ TNF-α levels
	Adult rats (i.c.v. injection)		↑ Cognitive performance
Eubanks et al., 2006	Aβ_1−40_	Δ^9^-THC	AchE inhibition
			↓ Aβ aggregation
			(No direct demonstration of CB_2_ involvement)
Esposito et al., 2006	Aβ_1−42_	WIN55,212-2	= iNOS levels
	C6 rat glioma cells	JWH015	= NO production
	PC12 neurons	SR144528	= Phosphorylated tau levels
Esposito et al., 2007	Aβ_1−42_	JWH-015	↑ Aβ-induced astrocytic proliferation (CB_2_ agonist)
	C6 rat glioma cells	SR144528	↓ Aβ-induced astrocytic proliferation (CB_2_ antagonist)
	Adult rats (cortical injection)		
Tolón et al., 2009	Aβ_1−42_	JWH-015	↑ Aβ plaque removal
	THP1 human macrophages	SR144528	↑ Aβ Phagocytosis
	U373 human astrocytoma		
	Human AD tissue sections		
Martín-Moreno et al., 2011	Aβ_1−42_	JWH-133	↓ ATP-induced increase in [Ca^2+^]_i_
	N13 and BV-2 microglial cells	WIN55,212-2	↑ Microglia migration
	Rat primary microglia culture	HU-308	↓ NO production
	Adult mice (i.c.v. injection)	SR144528	↑ Cognitive performance (no direct demonstration of CB_2_ involvement)
			↓ TNF-α and IL-6 expression (no direct demonstration of CB_2_ involvement)
Fakhfouri et al., 2012	Aβ_1−42_	WIN55,212-2	↑ Cognitive performance
	Adult rats (hippocampal injection)	SR144528	↓ TNF-α and nuclear NF-kB levels
			↓ Active caspase 3 levels and TUNEL-positive neurons
Martín-Moreno et al., 2012	TgAPP-2576 mice	JWH-133	↑ Cognitive performance
		WIN55,212-2	↑ Glucose uptake in brain
			↓ Microglial response to Aβ
			↓ Aβ deposition
			↓ TNF-α and COX-2 levels
			↑ Aβ transport across choroid plexus
Aso et al., 2013	APP/PS1 mice	JWH-133	↑ Cognitive performance
			↓ Microglial response to Aβ
			↓ Pro-inflammatory cytokines (IL-1β, IL-6, TNF-α, and IFN-γ)
			↓ Tau hyperphosphorylation around plaques
			↓ Oxidative stress damage around plaques
Wc et al., 2013	Aβ_1−40_	MDA7	↓ Expression of microglia and astrocyte markers
	Adult rats (hippocampal injection)		↓ Secretion of interleukin-1β
			↓ Upsurge of CB_2_ receptors
			↑ Aβ clearance
			↑ Synaptic plasticity
			↑ Cognitive performance
Bachmeier et al., 2013	Primary human brain microvascular	CB13	↑ Aβ transport across blood brain barrier
	endothelial cells	AM630	
	Adult mice (Caudate putamen injection)		
Chen et al., 2013	5xFAD APP mice	Δ^9^-THC	↓ Aβ deposition
			↓ Number of degenerated neurons
			(No direct demonstration of CB_2_ involvement)
Janefjord et al., 2014	Aβ_1−42_	JWH-015	↓ Aβ fibrillisation (no direct demonstration of CB_2_ involvement)
	Neuroblastoma SH-SY5Y cells	Δ^9^-THC	= Cell viability after Aβ_1−42_ exposure
	BV-2 microglial cells		↑ Cell viability after LPS exposure
Cao et al., 2014	N2a/APPswe cells	Δ^9^-THC	↓ Aβ levels
			↓ Aβ aggregation
			↓ Tau phosphorylation
			↑ Mitochondria function
			(No direct demonstration of CB_2_ involvement)
Aso et al., 2015	APP/PS1 mice	Δ^9^-THC	↑ Cognitive performance
			↓ Astroglial response to Aβ
			(No direct demonstration of CB_2_ involvement)
Köfalvi et al., 2016	TgAPP-2576 mice	JWH-133	↑ Glucose uptake in brain
		WIN55,212-2	
		AM630	

**Table 2:** **Evidence about the role of CB_**2**_ receptors in AD obtained from genetically modified mice**.

**References**	**Animal model**	**AD-related characteristics**
Chen et al., 2012	5xFAD/CB_2_(−/−)	= Effect of a MAGL inhibitor on reducing astrocytes
		Around plaques
Koppel et al., 2014	J20 APP/CB_2_(−/−)	↑ Soluble Aβ_1−42_
		↑ Plaque deposition
		↓ Total tau
		↑ Microglia associated to plaques
Schmöle et al., 2015	APP/PS1/CB_2_(−/−)	= Cognitive performance
		= Plaque deposition
		↓ Concentrations of soluble Aβ_1−40_and Aβ_1−42_
		↓ Microglial cells and infiltrated macrophages
		↓ Levels of pro-inflammatory chemokines and cytokines
Aso et al., 2016	APP/PS1/CB_2_(−/−)	= Cognitive performance
		= Cognitive improvement induced by Δ^9^-THC+CBD
		↑ Soluble Aβ_1−40_
		↑ Plaque deposition
		= Tau phosphorylation around plaques
		↓ Effect of Δ^9^-THC+CBD on reducing microglia around plaques

### Anti-inflammatory effects of CB_2_ receptor activity

Inflammation is common in most neurodegenerative diseases including AD, and it may contribute to progressive neuronal damage. Microglia play a major role in neuroinflammation. Activated microglia produce cytokines and mediators of inflammatory response which, in combination with neurons and astrocytes, create a complex cytokine cycle with deleterious consequences in brain when sustained over time (Heneka et al., 2014; McGeer and McGeer, 2015). CB_2_ receptors, mainly expressed in microglia, inhibit microglia-mediated neurotoxicity by reducing the production of pro-inflammatory molecules and by modulating macrophage migration in several pathological conditions (Cabral and Griffin-Thomas, 2009). In addition, CB_2_ activity facilitates the transformation of microglial cells from the M1 to M2 phenotype which is suggested to favor phagocytosis and reparative mechanisms (Mecha et al., 2015). As summarized in Table 1, a number of studies have shown anti-inflammatory effects of CB_2_ agonists in different models of AD. Thus, *in vitro* experiments have demonstrated that the selective agonists JWH-015, JWH-133, and HU-308, and the mixed CB_1_–CB_2_ receptor agonists WIN55,212-2 and HU-210 reduce the release of pro-inflammatory cytokines in microglial cell cultures exposed to different species of the toxic Aβ peptide (Ehrhart et al., 2005; Ramírez et al., 2005; Martín-Moreno et al., 2011). These findings may be the result of CB_2_ agonists reducing microglial activation by decreasing intracellular calcium concentration, as demonstrated in microglial cell cultures (Martín-Moreno et al., 2011). Moreover, JWH-133 and WIN55,212-2 promote microglial migration, which facilitates the phagocytosis of aggregated Aβ (Martín-Moreno et al., 2011). CB_2_ agonist JWH-015 facilitates Aβ-induced astrocytic proliferation in cell culture which participates in the inflammatory process as well (Esposito et al., 2007). These findings have been corroborated *in vivo* by the administration of selective CB_2_ and mixed CB_1_–CB_2_ receptor agonists to rats and mice inoculated with Aβ into the brain, resulting in reduced levels of several pro-inflammatory cytokines and decreased microglia reactivity to the Aβ insult (Ramírez et al., 2005; Esposito et al., 2007; Martín-Moreno et al., 2011; Fakhfouri et al., 2012; Wu et al., 2013). In some cases, the specificity of CB_2_-induced effects has been demonstrated by the co-administration of the selective CB_2_ antagonist SR144528 (Esposito et al., 2007; Martín-Moreno et al., 2011; Fakhfouri et al., 2012). Moreover, transgenic mice bearing APP mutations linked to familial AD exhibit a reduction in the number of activated microglial cells surrounding Aβ deposits and in the levels of pro-inflammatory cytokines after chronic treatment with the selective CB_2_ receptor agonist JWH-133 (Martín-Moreno et al., 2012; Aso et al., 2013). Considering that systemic inflammation may exacerbate the progression of AD (Lim et al., 2015) and that CB_2_ receptor is highly expressed in the peripheral immune system (Atwood and Mackie, 2010), it can be speculated that systemic CB_2_-driven actions may be also beneficial in AD.

Genetic models designed to unravel the role of CB_2_ receptors in AD progression have produced divergent findings regarding inflammatory responses (Table 2). A significant increase in the number of activated microglia associated with plaques has been reported in J20 APP transgenic AD mice lacking, in addition, the CB_2_ receptor (Koppel et al., 2014). Knocking down CB_2_ receptor gene in APP/PS1 mice results in a reduction of microglia reactivity and in the levels of pro-inflammatory chemokines and cytokines (Schmöle et al., 2015; Aso et al., 2016). Inhibition of monoacylglycerol lipase (MAGL), one of the main enzymes responsible for endocannabinoids degradation, results effective at reducing astroglial reaction to amyloid plaques in 5xFAD mice lacking CB_2_ receptor (Chen et al., 2012). However, the effect induced by the combination of Δ^9^-THC+CBD is reduced in APP/PS1 mice knockout for CB_2_ receptor (Aso et al., 2016). Divergent results may be related to the differing genetic backgrounds of mouse models, but in any case they point to a role for CB_2_ receptors in the control of microglial and inflammatory responses to Aβ insults.

### Modulation of Aβ and hyper-phosphorylated tau processing

A number of studies have proposed a direct role for CB_2_ receptors in the modulation of Aβ peptide levels in brain. Most of them suggest the participation of CB_2_ receptors in Aβ clearance rather than in Aβ production and aggregation. In this sense, activation of CB_2_ receptors with the specific agonist JWH-015 facilitates Aβ phagocytosis by human macrophages in brain sections obtained from AD cases (Tolón et al., 2009) and by microglia in cell culture (Ehrhart et al., 2005). Similarly, MDA7, another potent synthetic CB_2_ agonist, promotes Aβ clearance in the brains of Aβ-injected rats (Wu et al., 2013). JWH-133 and WIN55,212-2 favor Aβ transport through the choroid plexus *in vitro* (Martín-Moreno et al., 2012). The facilitation of Aβ clearance across the blood brain barrier has also been demonstrated using the synthetic CB_1_–CB_2_ receptor agonist CB13 in *in vitro* and *in vivo* models (Bachmeier et al., 2013). These findings may explain, at least in part, the reduction in Aβ levels in APP transgenic mice after chronic treatment with the agonists JWH-133 and WIN55,212-2 (Martín-Moreno et al., 2012). A few reports have also suggested a direct effect of the mixed CB_1_–CB_2_ agonist Δ^9^-THC on the reduction of Aβ aggregation (Eubanks et al., 2006; Cao et al., 2014; Janefjord et al., 2014) and on the promotion of Aβ degradation (Chen et al., 2013). However, demonstration of a direct involvement of CB_1_ or CB_2_ receptors is lacking in these studies.

Further evidence of CB_2_ participation in Aβ processing derives from the study of AD models with genetic deletion of this receptor (Table 2). Two of the three models had increased soluble Aβ levels and increased numbers of amyloid plaques in adult mouse brains (Koppel et al., 2014; Aso et al., 2016). In the case of APP/PS1 mice lacking CB_2_ receptors, the increased Aβ deposition observed may be related to the reduced microglial reaction in their brains (Aso et al., 2016), considering the role of CB_2_ activity in promoting microglial-induced Aβ phagocytosis (Ehrhart et al., 2005; Tolón et al., 2009). These observations reinforce the hypothesis that CB_2_ receptors facilitate Aβ clearance whereas their absence results in greater Aβ accumulation in brain. However, a slight reduction in soluble Aβ and plaque content has been reported in aged AD mice lacking CB_2_ receptors (Schmöle et al., 2015), suggesting that CB_2_ receptor participation in Aβ processing may vary along with the progression of the neurodegenerative process.

A role for CB_2_ receptors in the modulation of tau hyper-phosphorylation has also been proposed. Early studies performed in cell cultures demonstrated that the mixed CB_1_–CB_2_ agonist WIN55,212-2 inhibited tau protein hyper-phosphorylation in Aβ-stimulated PC12 neuronal cells, but that this effect was mediated mainly by CB_1_ receptors (Esposito et al., 2006). Moreover, a specific CB_2_ agonist failed to modify tau hyper-phosphorylation in the same experimental conditions (Esposito et al., 2006). Δ^9^-THC, a mixed CB_1_–CB_2_ agonist, is able to reduce tau phosphorylation in N2a/APPswe cells (Cao et al., 2014) but no direct evidence has been found about the specific involvement of CB_2_ receptors in such effect. It is worth noting that *in vivo* experiments have demonstrated that chronic treatment with the specific CB_2_ agonist JWH-133 significantly reduces tau hyper-phosphorylation at the Thr181 site in the vicinity of Aβ plaques in APP/PS1 mice (Aso et al., 2013). This effect may be explained by concomitant decreased expression of active forms of GSK3β, p38 and SAPK/JNK in the vicinity of Aβ plaques in JWH-133-treated APP/PS1 mice (Aso et al., 2013). In contrast, no difference in tau hyper-phosphorylation at site Thr181 was observed in APP/PS1 mice lacking CB_2_ receptors (Aso et al., 2016), suggesting that the activation of these receptors may avoid tau phosphorylation but their absence does not alter the process of tau phosphorylation. Yet J20 APP mice knocked out for the CB_2_ receptor gene show decreased levels of total tau protein without modifications of its phosphorylation state (Koppel et al., 2014). Considering all these observations, it is clear that the role of CB_2_ receptors in tau processing requires further investigation, as the available information is variegated and not conclusive.

### Other effects: neuronal survival, anti-oxidative, glucose metabolism, and cognition

Targeting CB_2_ receptors produces additional benefits in AD. Thus, CB_2_ receptor agonists promote cell survival in the face of Aβ insults in *in vitro* and *in vivo* models (Ramírez et al., 2005; Fakhfouri et al., 2012; Chen et al., 2013; Janefjord et al., 2014). Moreover, anti-oxidant effects have been reported for compounds activating CB_2_ receptors. Specifically, two studies have demonstrated that specific CB_2_ agonists reduce the production of free radical NO induced by Aβ exposure in microglial cell culture (Ehrhart et al., 2005; Martín-Moreno et al., 2011), although these results have not been replicated in a glioma cell line (Esposito et al., 2006). *In vivo* experiments also show that activation of CB_2_ receptors reduces oxidative stress damage and promotes anti-oxidative stress responses; chronic treatment with JWH-133 reduces hydroxynonenal adducts derived from lipid peroxidation and enhances the levels of superoxide dismutase 1 and superoxide dismutase 2 in the vicinity of plaques in APP/PS1 mice (Aso et al., 2013). The mechanisms by which CB_2_ receptors mediate these anti-oxidant effects remain elusive. It has been reported that the CB_1_–CB_2_ agonist Δ^9^-THC improves mitochondrial function (Cao et al., 2014), thus presumably contributing to a reduction in the production of free radicals, but further study is needed to support this hypothesis. Additional benefits of the activation of CB_2_ receptors in AD may derive from the ability of these receptors to mediate glucose uptake in brain (Martín-Moreno et al., 2012; Köfalvi et al., 2016), which may counteract the well-known glucose metabolism deficit in AD brains (Mosconi et al., 2008; Cohen and Klunk, 2014).

More importantly, CB_2_ selective and CB_1_–CB_2_ mixed agonists prevent memory deficits in Aβ-injected rats and mice after chronic administration (Ramírez et al., 2005; Martín-Moreno et al., 2011; Fakhfouri et al., 2012; Wu et al., 2013) and improve cognitive performance in two different transgenic mouse models of AD (Martín-Moreno et al., 2012; Aso et al., 2013). The mechanisms of action underlying cognitive improvement are assumed to be multiple and likely related mainly to the capacity of CB_2_ receptors to mitigate the harmful effects of several molecules produced in AD brains. In fact, AD-like mice lacking CB_2_ receptors display the same cognitive performance as the corresponding transgenic control mice (Schmöle et al., 2015; Aso et al., 2016), suggesting that CB_2_ receptors may not play a direct role on cognition.

## Conclusions and future perspectives

**Figure 1:**
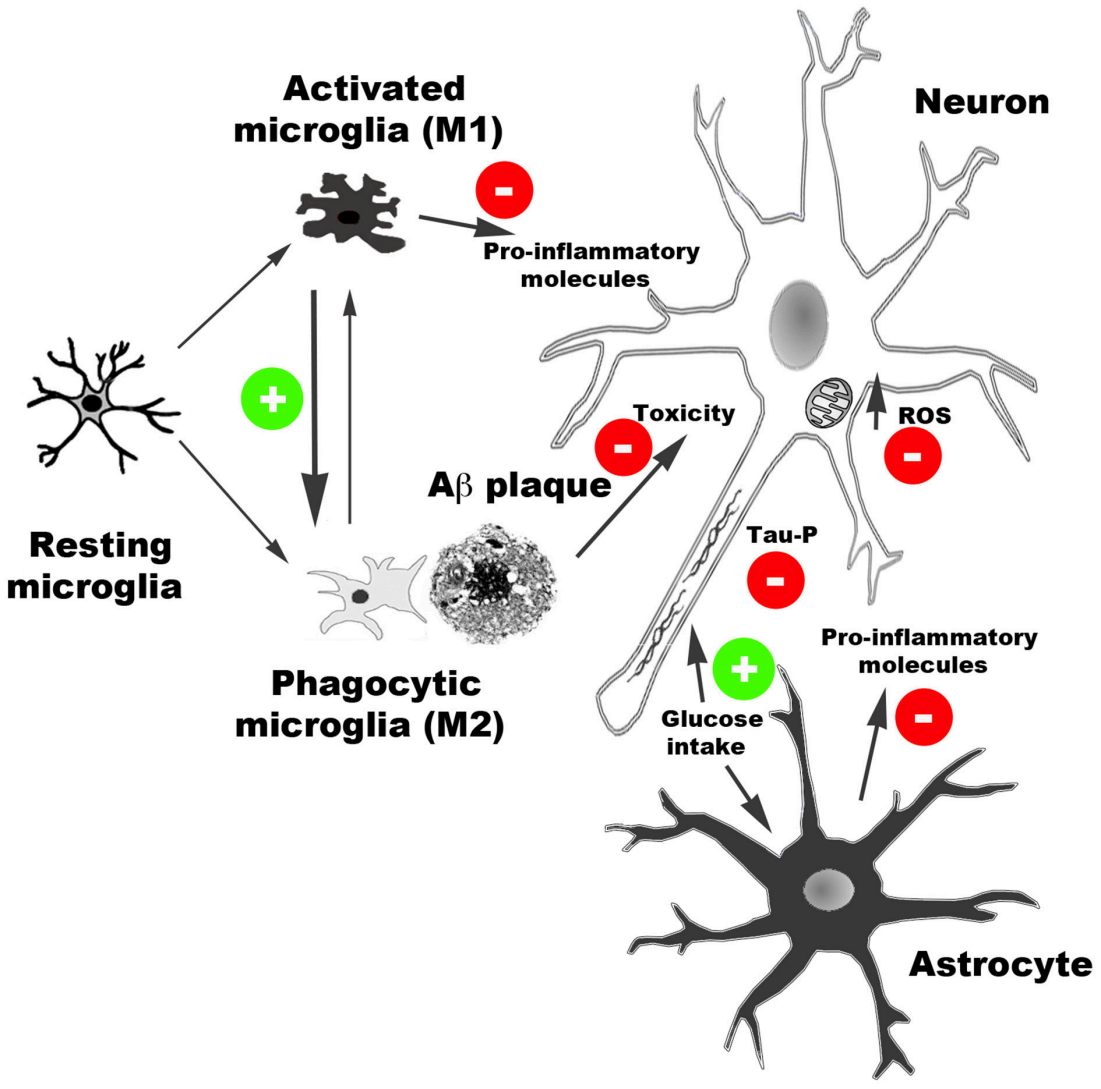
**Schematic representation of main effects of CB_**2**_ receptor activation reported in AD models**. CB_2_ receptor agonists reduce the release of pro-inflammatory molecules, facilitate Aβ clearance by promoting microglia phagocytic phenotype, reduce Aβ neurotoxicity, and facilitate glucose uptake. Moreover, CB_2_-mediated activity reduces oxidative stress damage produced by reactive oxidative species (ROS) and tau hyper-phosphorylation.

Taken together, the experimental observations discussed in the present review indicate that AD induces CB_2_ receptor expression and that targeting CB_2_ receptors has beneficial effects in AD. Specifically, CB_2_ receptor agonists reduce inflammatory responses linked to Aβ production and deposition, facilitate Aβ clearance, increase cell viability in the presence of Aβ, and promote glucose uptake in brain. Moreover, CB_2_ activity likely reduces tau hyper-phosphorylation and oxidative stress damage caused by Aβ peptides (Figure 1). As a result of the combination of these effects, among others, CB_2_ receptor agonists induce cognitive improvement in AD models.

Considering the evidence of pleiotropic activity and lack of undesirable psychoactive effects of CB_2_ receptors, compounds acting on such cannabinoid receptors represent a promising therapy against AD. Nevertheless, there is still no information regarding the efficacy or toxicity in human beings of compounds specifically targeting CB_2_ receptors, which might exhibit some side effects such as immune suppression (Pertwee, 2005). For these reasons, progress toward clinical practice requires further investigation.

## Author contributions

EA and IF contributed equally to writing this review. Both authors give final approval of the text.

## Funding

The authors' work is supported by CIBERNED, Institute of Health Carlos III (Spanish Ministry of Economy and Competitiveness) and confunded by FEDER funds/European Regional Development Fund (ERDF) - a way to build Europe (PI14/00757 to IF).

### Conflict of interest statement

The authors declare that the research was conducted in the absence of any commercial or financial relationships that could be construed as a potential conflict of interest.
